# Orbital cellulits following cataract surgery under peribulbar anaesthesia

**DOI:** 10.3205/oc000024

**Published:** 2015-01-16

**Authors:** Chandoshi Mukherjee, Arijit Mitra, Bushra Mushtaq

**Affiliations:** 1 Birmingham Midland Eye Centre, City Hospital, Dudley Road, Birmingham, United Kingdom

**Keywords:** orbital cellulitis, peribulbar anaesthesia, complication

## Abstract

**Introduction:** Orbital cellulits following cataract surgery is extremely rare. We describe a case of orbital and facial cellulitis that occurred after routine cataract operation with peribulbar anaesthesia. There were no preoperative systemic or ocular risk factors for postoperative infection.

**Case description:** An 89-year-old man presented to eye casualty, the day after he underwent an uneventful phacoemulsification and posterior chamber lens implantation in the left eye under peribulabr anaesthesia, with soreness, swelling and reduced visual acuity (6/18) in the operated eye.

On initial presentation periorbital swelling was noted, the eye was minimally tender, intraocular pressure was raised at 28 mHg and fundoscopy was limited due to a hazy cornea. The patient was discharged on topical medication with a diagnosis of allergic reaction to postoperative drops.

The following day, the patient re-presented with worsening orbital swelling involving the left cheek. Ocular findings remained unchanged. CT scan revealed left orbit soft tissue swelling and a locule of air medial to the medial rectus. There were no signs of sinus infection or periosteal inflammation. A diagnosis of left orbital and facial cellulitis was made and the patient was treated with intravenous antibiotics.

**Discussion:** Our patient did not have any predisposing risk factors, therefore most likely cause of cellulitis is surgical trauma during administration of the peribulbar block.

This case illustrates the need for adequate skin preparation before the administration of peribulbar anaesthesia and minimal tissue trauma during the procedure.

## Introduction

Orbital cellulits following ophthalmic surgery is and is extremely rare following cataract surgery [1], [2], [3]. We describe a case of orbital and facial cellulitis that occurred after routine cataract operation with peribulbar anaesthesia. There were no preoperative systemic or ocular risk factors for postoperative infection. 

## Case report

An 89-year-old man underwent an uneventful phacoemulsification and posterior chamber lens implantation in the left eye. A peribulbar injection consisting of 2 ml of 2% lignocaine hydrochloride and 4 ml of 7.5 mg/ml of Bupivicaine hydrochloride along with 150 units of hyaluronidase was administered. A total of 8.5 ml was injected as a single medial canthus injection using a 25 gauge needle. Skin preparation with 5% povidone iodine was done prior to the block. No complications were noted during or after the peribulbar injection.

**Figure 1:**
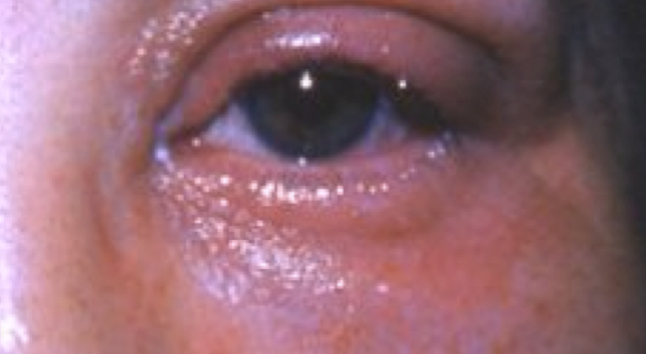
Periorbital swelling

The day after the surgery, the patient presented to the eye casualty with a sore, slightly swollen left eye (OS) and a visual acuity of 6/18 right eye (OD) and 4/60 OS. On examination, upper and lower lid edema was noted (Figure 1 (Fig. 1)) along with conjunctival injection and corneal stromal edema. Both anterior segments were deep and quiet. The eyeball was minimally tender. There was no proptosis. Full range of eye movements was preserved and pupils were equal and reactive. The intraocular pressure was raised at 28 mmHg. Examination of the left fundus was grossly normal although examination was difficult due to a hazy cornea. A diagnosis of possible allergic reaction to post operative drops was made and the patient was commenced on preservative free (PF) dexamethasone 0.1% and chloramphenicol 0.5% along with 4 mg on chlorpheneramine Three times a day orally. 

**Figure 2:**
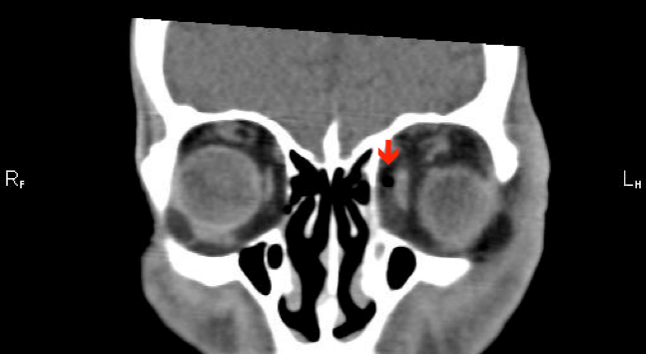
Coronal section of MRI scan showing air locule

**Figure 3:**
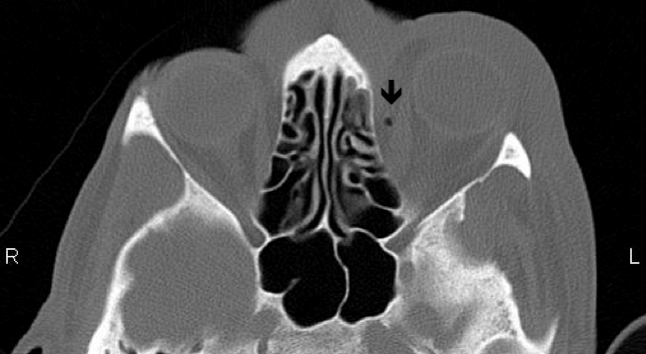
Transverse section of MRI scan showing air locule

The following day, the patient re-presented with worsening orbital swelling now involving the left cheek. On further questioning, it was noted that he had a history of sinusitis and had recently suffered from an episode of upper respiratory tract infection. Ocular findings remained unchanged. Computed tomography (CT) scan revealed left orbit soft tissue swelling outside the globe along with soft tissue changes consistent with inflammation and a locule of air in the orbital cavity, medial to the medial rectus (Figure 2 (Fig. 2), Figure 3 (Fig. 3)). There were no signs of sinus infection or periosteal inflammation (Figure 2 (Fig. 2), Figure 3 (Fig. 3)). C-reactive protein (CRP) was raised at 35. A diagnosis of left orbital and facial cellulitis was made. The patient was admitted and commenced on 2 g of intravenous ceftriaxone once a day and 400 mg of metronidazole three times a day. Blood cultures did not show any organisms. The celluitis responded well to treatment and he was discharged two days later with oral antibiotics. Two weeks later, the patient was reviewed in clinic. His best, corrected visual acuity was 6/12 OD and 6/9 OS. 

## Discussion

Orbital cellulitis as a complication of cataract surgery is rare [1], [2], [3]. It has been postulated that antiseptic skin preparation with alcohol or chlorohexidine may be inadequate; therefore it is recommended that povidone-iodine be used instead before administration of a peribulbar block [2], [4]. In our case as well as one, previously reported, skin preparation with povidone-iodine did not prevent infection. However, the povidone-iodine needs to be left for about 5–10 minutes in order to sterilize the surface [4]. Trauma during injection along with ecchymosis possibly aids the spread of infection, therefore an aseptic technique with minimal soft tissue trauma is recommended [1].

Predisposing risk factors for orbital cellulitis include sinusitis, dental or ear infection and trauma [3]. Even though our patient had a history of sinusitis the CT did not show inflammation within the sinuses. There was no history of dental or ear infection. Also, our patient was not diabetic or immunocompromised. Therefore in this case the most likely cause of cellulitis is surgical trauma during administration of the peribulbar block. 

Our patient had a unique presentation of associated facial cellulitis, which is worth noting. The presence of an air locule in the orbital cavity could be either due to injection of air from the needle during the peribulbar injection or due to anaerobic gas-forming organisms which were the cause of the infection. 

The rise in intraocular pressure was due to edema of the intraorbital tissue and increased venous pressure [2], [5].

Orbital cellulitis is usually treated with parenteral broad-spectrum antibiotics such as third or fourth generation cephalosporin’s and metronidazole to cover anaerobes [6]. Prompt recognition and treatment resulted in a favorable outcome in this case. Skin preparation 5–10 minutes before peribulbar anaesthesia should be performed in all cases in order to reduce the incidence of cellulitis, with minimal soft tissue trauma during peribulbar anaesthesia.

## Notes

### Competing interests

The authors declare that they have no competing interests.
